# Effectiveness of Anti-SARS-CoV-2 monoclonal antibodies in real-life: RNAemia and clinical outcomes in high-risk COVID-19 patients

**DOI:** 10.1371/journal.pone.0321356

**Published:** 2025-04-25

**Authors:** Matteo Domenico Marsiglia, Silvia Bianchi, Francesca Bai, Camilla Tincati, Emerenziana Ottaviano, Silvia Ancona, Giulia Marchetti, Elisa Borghi

**Affiliations:** 1 Department of Health Sciences, Università degli Studi di Milano, Milano, Italy; 2 Clinic of Infectious Diseases, San Paolo Hospital, ASST Santi Paolo e Carlo, Department of Health Sciences, Università degli Studi di Milano, Milano, Italy; University of North Dakota, UNITED STATES OF AMERICA

## Abstract

**Background:**

Anti-SARS-CoV-2 neutralizing monoclonal antibodies (mAbs) have been shown to have clinical benefits in treating high-risk patients with mild-moderate COVID-19. SARS-CoV-2 RNA in serum (RNAemia), is usually associated with severe disease and deaths. This study evaluates real-life data on the effectiveness of mAbs therapies against SARS-CoV-2 infections by different viral variants, particularly in the presence of RNAemia, focusing on clinical outcomes.

**Methods:**

From March 2021 to May 2022, high-risk patients with PCR-confirmed mild-moderate COVID-19 were enrolled at the Clinic of Infectious Diseases, San Paolo Hospital in Milan. Patients received Bamlanivimab/Bamlanivimab + Etesevimab/Casirivimab + Imdevimab/Sotrovimab based on Agenzia Italiana del Farmaco (AIFA) guidelines and prevalent SARS-CoV-2 variants. Nasopharyngeal swabs (NPS) and plasma samples were collected at infusion (t0) and after 7 days (t1). NPS viral loads and RNAemia were quantified using RT-qPCR, and variant typing was conducted. Clinical outcomes were evaluated, including time to symptom resolution and adverse effects.

**Results:**

Among 176 enrolled patients, treatment efficacy was observed in 96.6% with a median time to symptom resolution of 12 days (IQR 10–19). Viral load significantly decreased in both NPS and plasma by day 7 post-treatment (p<0.001). At t0, RNAemia was present in 61.9% of patients and NPS viral loads were higher in patients with RNAemia (p=0.002). However, after treatment, no significant differences in viral loads and times to symptom resolution were noted between patients with and without RNAemia. Omicron-infected patients exhibited higher plasma viral loads compared to Alpha and Delta variants (p<0.001) and the presence of RNAemia was significantly associated with Omicron (p<0.001). Vaccinated patients achieved faster recovery regardless of variant type (p=0.001).

**Conclusion:**

Early administration of anti-SARS-CoV-2 mAbs in high-risk patients significantly reduced viral loads in NPS and plasma and improved clinical outcomes. Despite the presence of RNAemia, these tailored mAb therapies led to favorable recovery times and minimal adverse effects.

## Introduction

Since the onset of the SARS-CoV-2 pandemic, institutions and researchers have focused on avoiding the virus worldwide spread and reducing the related mortality. Diverse therapeutic strategies have been proposed to treat COVID-19 patients, predominantly encompassing antiviral agents, anti-IL-6 receptor antibodies, Janus kinase inhibitors, and neutralizing antibody therapies [1–3]. The advent and use of monoclonal antibodies (mAbs) have revolutionized the treatment of various pathologies such as cancer, organ transplants, and autoimmune, cardiovascular, respiratory and neurological diseases [4,5].

While vaccines remain the best strategy to prevent severe COVID-19 disease, anti-SARS-CoV-2 mAbs have shown efficacy and good tolerability in outpatients at high-risk of severe disease with mild-moderate symptoms. Available mAbs, designed to target the Spike (S) protein of the SARS-CoV-2 virus, aim at impeding the virus cellular entry through their interaction with the ACE2 receptor [6,7].

From the earliest days of the pandemics emergence to the present, different genetic sequences have been described from around the world, revealing the variants of SARS-CoV-2. Different mutations, particularly within the RdRp gene and the coding sequence for the Receptor Binding Domain (RBD) of the S protein that modulate virus transmissibility and antibody-mediated neutralization, have been described [8]. As the virus continues to mutate, reducing antibodies effectiveness against distinct variants, the therapeutic employment of mAbs has required adaptation to match the evolving virus [6,9,10].

Eight distinct mAbs (namely Bamlanivimab, Etesevimab, Casirivimab, Imdevimab, Sotrovimab, Cilgavimab, Tixagevimab, and Regdanvimab) have received approval for clinical use in Italy and in Europe [11–16].

Bamlanivimab, a humanised IgG-neutralising antibody targeting the RBD of the S protein, was the first mAb approved for clinical application. However, owing to viral mutations, Bamlanivimab’s potency against the Beta, Gamma, and Delta variants waned [8]. As a result, monotherapy authorization for Bamlanivimab was rescinded, making way for a combination therapy involving Bamlanivimab and Etesevimab [17]. The latter targets an alternate epitope on the S protein, and the combination of these mAbs was demonstrated to neutralize the Alpha and Delta variants [8,10].

Within the therapeutic landscape, Casirivimab and Imdevimab stand as a pair of non-competing mAbs, each binding distinct epitopes on the RBD of the S protein. This dual regimen exhibits efficacy against a range of variants, including Alpha, Beta, Gamma, and Delta. However, the Omicron variant has demonstrated a capacity to evade neutralization by the Bamlanivimab/Etesevimab and Casirivimab/Imdevimab combinations, prompting a temporary suspension of these treatments [18,19]. The combination of Casirivimab/Imdevimab has received approval from Agenzia Italiana del Farmaco (AIFA) with two different dosages: 1200 mg and 4000 mg. Casirivimab/Imdevimab at 1200 mg has been authorized for non-hospitalised patients, while the 4000 mg dosage is intended for hospitalised patients [20].

Sotrovimab, a derivative of an antibody stemming from an individual previously infected with SARS-CoV in 2003, uniquely binds an RBD epitope shared between SARS-CoV and its contemporary counterpart, SARS-CoV-2 [8]. Notably, Sotrovimab has exhibited efficacy against the Beta, Delta, and Omicron variants [6,21]. Sotrovimab showed a decreased in vitro neutralization against Omicron BA.2; however, real-life observational studies have confirmed its efficacy in reducing clinical progression on Omicron BA.1 and BA.2. The translation of the in vitro data in clinical effectiveness in fact remains uncertain, also considering the potent Fc-effector functions of the monoclonal antibody that include antibody-dependent cellular cytotoxicity and antibody-dependent cellular phagocytosis [22,23].

SARS-CoV-2 RNA presence in bloodstream, a condition also known as RNAemia, has been suggested as a valid biomarker for plasma viremia [24]. RNAemia has been correlated with disease severity, admission to intensive care units, mortality in COVID‐19 cases, and may act as a clinical predictor. Hence, the spread of SARS-CoV-2 into the bloodstream could be a crucial event in the pathogenesis of COVID-19, triggering an inflammatory response that can lead to multiorgan failure. Blood donations have revealed that the tendency to enter the bloodstream is variant-dependent, with the Delta and Omicron variants displaying the higher values [25,26]. RNAemia underscores the potential for severe outcomes in COVID-19 patients, highlighting the importance of monitoring viral presence in the bloodstream as a critical aspect of clinical management [27,28].

Data from real-life settings seem to confirm the optimal safety profile and the reduction in hospitalisation rates and mortality following mAbs treatments [29]. Here, we present real-life data concerning the effectiveness and safety of anti-SARS-CoV-2 mAbs in a cohort of high-risk patients in Milan. Our focus was to explore the potential relationships between nasopharyngeal viral load and SARS-CoV-2 RNAemia at baseline and 7th day post-treatment according to the infecting variants.

## Materials and methods

### Study design and population

From 18 March 2021 to 23 May 2022, high-risk subjects with mild-moderate COVID-19, PCR-confirmed, were consecutively enrolled at the Clinic of Infectious Diseases, San Paolo hospital in Milan. High-risk patients were defined according to AIFA indications for treatment with mAbs, i.e., age ≥65 yrs, BMI≥30, cardio-cerebrovascular diseases, chronic lung diseases, diabetes, immunodeficiencies, chronic kidney failure and dialysis, chronic liver diseases. We collected demographic data (age, sex), risk factors for the development of severe disease, blood tests (C-Reactive Protein, blood count, transaminases, creatinine), sO_2_ at admission, and any concomitant therapies.

Patients were treated with Bamlanivimab 700mg (BAM), Bamlanivimab 700mg + Etesevimab 1400mg (BAM/ETE), Casirivimab 1200mg + Imdevimab 1200mg (CAS/IMD 1200mg), Casirivimab 4000mg + Imdevimab 4000mg (CAS/IMD 4000mg), or Sotrovimab 500mg (SOT) in a dedicated ID outpatient service or during hospitalisation in the ID Clinic (San Paolo hospital) following the AIFA guidelines and in relation to prevalent SARS-CoV-2 variants [11–14,20]. We collected nasopharyngeal swab (NPS) and plasma at infusion (t0) and after 7 days (t1). The median number of days from symptoms onset to clinical recovery (defined by resolution of all signs/symptoms of COVID-19) and the outcome (defined as clinical recovery versus all-reasons death) was evaluated.

Identifiable data from participants (e.g., age, gender, medical information) were saved in encrypted password-protected files. Investigators had access to these data only for research purpose from June 8^th^, 2022. The study has received approval by the Ethic Committee Milano Area 1 (no. 0000677, 01/04/2020) and all the subjects provided written informed consent to participate in the study. The study has been conducted according to the World Medical Association and the Declaration of Helsinki.

### SARS-CoV-2 RNA quantification and variant analysis

RNA was extracted from paired NPS and plasma samples with the commercial method QIAamp ViralRNA Mini Kit (QIAGEN, Germany) by following the manufacturer’s instructions. Five µl of RNA was used for RT-qPCR using COVID-19 HT Screen (Clonit, Italy) and SARS-CoV-2 viral load was measured on paired NPS and plasma. For absolute quantification, serial dilutions (1:10) of the plasmid 2019-nCoV_Positive Control (Integrated DNA Technologies, Inc., USA) were used to generate a standard curve. The cut off for cycle threshold (Ct) values ≤ 38 was used for defining positive samples for both NPS and plasma samples. The assay was performed in duplicate for each sample and a control well was included as a negative control. Quantification of the human *RPP30* gene was performed to determine the quality of RNA extraction.

Variant typing was conducted on RNA extracted from NPS samples using the Allplex^TM^ SARS-CoV-2 Variants I and II assays developed by Seegene Inc. (Republic of Korea). The Seegene Variants I assay is specifically designed to identify the SARS-CoV-2 RdRP gene and Alpha, Beta, Gamma, Omicron and Mu variants. The Seegene Variants II assay is designed to detect Beta, Gamma, Delta and Omicron. The assays were performed according to the manufacturer’s instructions.

### Statistical analyses

Continuous variables were expressed as median (interquartile range, IQR), while categorical variables as number, n (percentage, %). Mann-Whitney U and Kruskal-Wallis tests were used for comparisons between groups for continuous variables, and the chi-square test was applied for categorical variables. The correction of multiple comparisons was adjusted by the Dunn’s test. P values less than 0.05 were considered statistically significant. Statistical analyses were conducted using R 4.3.1.

## Results

From March 2021 to May 2022, we enrolled 176 high-risk patients who met the enrollment criteria. The monoclonal therapy was administered within 4 days (median, IQR 2–6) from the onset of the symptoms. At infusion (t0) and after 7 days (t1) paired NPS and plasma were collected and analyzed. A summary of demographic, clinical and virological characteristics of the enrolled population is reported in Table 1.

**Table 1:** Demographic, clinical and virological characteristics of enrolled patients compared with infecting variants.

Characteristics	Total	Alpha	Delta	Omicron	Not Typed	p value
	N 176	N 56	N 74	N 35	N 10	
**Age (years), median (IQR)**	66 (51-76)	64 (53-76)	67 (51-80)	65 (50-75)	61 (48-77)	0.78
**Male sex, n (%)**	109 (61.9)	36 (64.3)	41 (55.4)	25 (71.4)	6 (60.0)	0.421
**Risk factor, n (%):**	72 (40.9)	20 (35.7)	34 (46.0)	12 (34.3)	6 (60.0)	0.293
**One risk factor**	65 (36.9)	26 (46.4)	20 (27.0)	14 (40.0)	3 (30.0)	
**Two risk factors**	39 (22.2)	10 (17.9)	20 (27.0)	9 (25.7)	1 (10.0)	
**Three or more risk factors**						
**Not hospitalised, n (%)**	133 (75.6)	49 (87.5)	42 (56.8)	32 (91.4)	9 (90.0)	<0.001
**Hospitalised (not COVID-19 related), n (%)**	16 (9.1)	7 (12.5)	7 (9.5)	1 (2.9)	1 (10.0)	
**Hospitalized (COVID-19), n (%)**	27 (15.3)	0	25 (33.8)	2 (5.7)	0	
**mAbs therapies, n (%):**	8 (4.5)	8 (14.3)	/	/	/	<0.001
**BAM**	60 (34.1)	24 (42.9)	14 (18.9)	15 (42.9)	6 (60.0)	
**BAM/ETE**	66 (37.5)	24 (42.9)	35 (47.3)	4 (11.4)	3 (30.0)	
**CAS/IMD 1200 mg**	25 (14.2)	/	25 (33.8)	/	/	
**CAS/IMD 4000 mg**	17 (9.7)	/	/	16 (45.7)	1 (10.0)	
**SOT**						
**Unvaccinated, n (%)**	92 (52.3)	51 (91.1)	26 (35.1)	9 (25.7)	5 (50.0)	<0.001
**Vaccinated, n (%)**	84 (47.7)	5 (8.9)	48 (64.9)	26 (74.3)	5 (50.0)	<0.001
**One dose**	9 (10.7)	5 (100.0)	1 (2.1)	3 (11.5)	/	
**Two doses**	54 (64.3)	/	42 (87.5)	10 (38.5)	3 (60.0)	
**Three doses**	21 (25.0)	/	5 (10.4)	13 (50.0)	2 (40.0)	
**Viral load in NPS (log**_**10**_ **copies/mL) at t0, median (IQR)**	8.5 (7.2 -9.4)	8.7 (6.8 -9.4)	8.5 (7.0 -9.4)	9.0 (7.9 -9.6)	5.9 (5.4 -8.1)	0.006
**Viral load in NPS (log**_**10**_ **copies/mL) at t1, median (IQR)**	5.6 (4.3-7.3)	6.1 (3.9-7.5)	6.0 (4.6-7.3)	5.5 (4.7-6.9)	5.4 (4.4-6.6)	0.764
**Viral load in plasma (log**_**10**_ **copies/mL) at t0, median (IQR)**	2.8 (0-4.1)	1.2 (0-3.0)	2.7 (0-4.1)	4.1 (3.5-5.8)	1.9 (0-4.4)	<0.001
**Viral load in plasma (log**_**10**_ **copies/mL) at t1, median (IQR)**	0.3 (0.0-3.6)	0.0 (0.0-0.0)	0.1 (0.0-3.8)	3.7 (2.9-4.2)	1.3 (0.0-3.2)	<0.001

### Virological characterisation of SARS-CoV-2 infections

**Fig 1:**
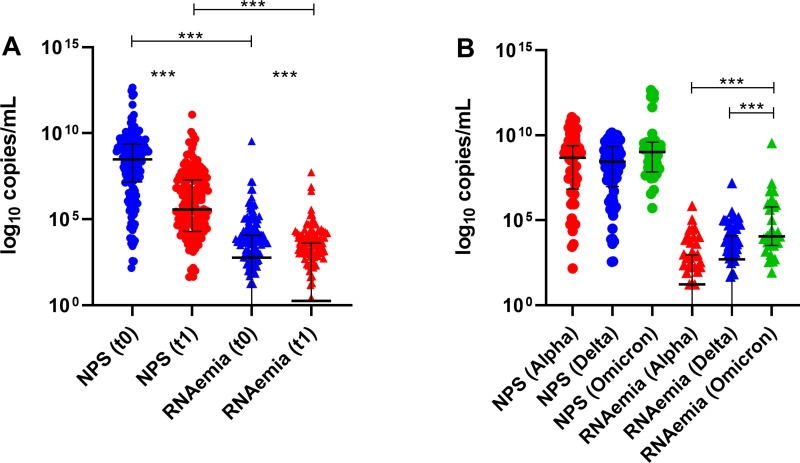
A) Viral load in NPS and RNAemia at t0 and t1; B) Viral load in NPS and RNAemia considering the infecting variant at t0. ***: p value ≤ 0.001.

Viral load in NPS was higher than in plasma samples (RNAemia) (p<0.001) at both time points (t0 and t1), with a significant reduction (p<0.001) after 7 days (Fig 1.A). At t0, 109 (61.9%, 109/176) patients with RNAemia displayed a higher viral load in NPS (8.9 log_10_ copies/mL, IQR 7.5–9.6) compared to those (38,1%, 67/176) without RNAemia (8.0 log_10_ copies/mL, IQR 6.1–9.0), p=0.002. At t1, this difference was not observed between the two groups (6.1 log_10_ copies/mL, IQR 4.4–7.5 vs 5.3 log_10_ copies/mL, IQR 4.1–6.9), p=0.102. Moreover, the viral load in NPS significantly decreased within the two groups (t0 vs t1, p<0.001).

SARS-CoV-2 variant typing was conducted on all RNA extracted from NPS. Ten out of 176 (5.7%) were not typeable. Alpha variant was detected in 56 out of 166 (33.7%) samples (March-May 2021), Beta in 1 (0.6%) sample (March 2021), Delta in 74 (44.6%) samples (May 2021 - January 2022), and Omicron in 35 (21.1%) samples (December 2021 - May 2022). Patients with RNAemia were mostly affected by Omicron variant (p<0.001). No significant difference in NPS viral load was found across patients infected with the various variants (p=0.098), while higher RNAemia was observed in Omicron-infected patients (p<0.001) (Fig 1.B).

Eighty-four out of 176 (47.7%) patients were vaccinated. 88.1% (74/84) of them were infected by Omicron or Delta variants (Table 1). Viral RNA was detectable in 71.4% (60/84) of plasma from vaccinated patients. The viral load in NPS at t0 showed no statistical differences between vaccinated (8.5 log_10_ copies/mL, IQR 6.9–9.6) and unvaccinated patients (8.4 log10 copies/mL, IQR 7.2–9.2), p=0.554. RNAemia was higher in vaccinated patients (3.5 log_10_ copies/mL, IQR 0.0–4.2) compared to unvaccinated (1.9 log10 copies/mL, IQR 0.0–3.7), p=0.002.

### Clinical outcome

Treatment showed efficacy in 96.6% of patients and clinical recovery was obtained in 12 days (median, IQR 10–19). Considering the clinical outcome correlated with mAbs treatments, we observed a median time to symptom resolution of 17 days with BAM (IQR 12–24), 14 days with BAM/ETE (IQR 10–21), 13 days with CAS/IMD 1200 mg (IQR 10–20), 11 days with CAS/IMD 4000mg (IQR 8–19), and 10 days with SOT (IQR 9–11). Total adverse effects were few and mainly fever or nausea (10.2%); no serious adverse effects were observed.

Patients with RNAemia at t0 had a median clinical recovery of 12 days (IQR 10–20), while those without RNAemia recorded a median recovery time of 13 days (IQR 9–18) (p=0.932). The median times to symptom resolution varied among different variants: 18 days for Alpha (IQR 14–24), 11 days for Delta (IQR 10–18), and 10 days for Omicron (IQR 9–11). Patients affected by Alpha variant showed a longer clinical recovery compared to both Delta and Omicron patients (p< 0.001). The distribution of viral variants in patients with and without RNAemia and clinical outcome is shown in Table 2.

**Table 2:** Patients with or without detectable RNAemia at t0, infecting variants and recovery time.

Variants	With RNAemia		Without RNAemia		p value
	N (%)	Clinical recovery (median, IQR)	N (%)	Clinical recovery (median, IQR)	
Alpha, N=56	29 (51.8)	18 (11-22)	27 (48.2)	18 (15-28)	0.184
Delta, N=74	42 (56.8)	13 (10-19)	32 (43.2)	11 (8-12)	**0.005**
Omicron, N=35	33 (94.3)	10 (9-14)	2 (5.7)	26 (10-42)	0.400

Regardless of the infecting variant, vaccinated patients achieved clinical recovery more quickly (median 11 days, IQR 9–16) than non-vaccinated patients (median 16 days, IQR 11–22) (p=0.001). Clinical recovery was achieved by 95.2% in vaccinated and 97.8% in unvaccinated patients.

During the study, 6 patients (3.4%) with a median age of 87 (IQR 82–94) died within 12 days (IQR 8–21) from therapy onset. Therapy was administered 4 days after the onset of symptoms (IQR 2–5). Two patients were hospitalised for COVID-19, while the other 4 were hospitalised for different reasons. At t0, RNAemia was present in 4 of the 6 patients. Five out of 6 patients were positive for the Delta variant and one patient for an uncharacterized variant. The causes of death were COVID-19-related respiratory failure in 3 patients and cancer in the remaining 3.

## Discussion

In this study, individuals at high-risk of severe COVID-19 were treated with mAbs and monitored to investigate viral loads in NPS, RNAemia and clinical outcomes after treatments. One of the challenges of mAbs therapies was to counteract escape variants able to undermine their therapeutic efficacy [8,30]. Therefore, in response to this challenge, several therapies with mAbs have been developed during the pandemic. This study highlighted that real-time adapted therapies and the early treatment were effective in reducing disease progression and mortality in individuals at high-risk of severe illness [31,32]. Adverse effects were generally limited, with post-infusion fever and nausea/vomiting reported. Investigating the efficacy of treatments, viral load significantly decreased in both NPS and plasma samples following therapies.

The study was conducted between March 2021 and May 2022, during which four different SARS-CoV-2 variants (Alpha, Beta, Delta, Omicron) emerged. In this different epidemic scenario, patients affected by Alpha variant exhibited a longer recovery time compared to those with Delta and Omicron variants although an extended recovery period has also been associated with the Delta variant [33]. This is probably due to the low number of vaccinated patients (5.9%) affected by Alpha variant considering that our findings demonstrated that vaccinated patients achieve clinical recovery more rapidly than unvaccinated [34].

In agreement with previous studies [35–38], no significant differences in viral load in NPS across patients with different SARS-CoV-2 variants were observed in our cohort. In contrast, other studies reported a higher viral load in patients with Delta variant [39] or with Omicron [40], and others suggested that Omicron exhibits a lower viral load when compared to the Delta variant [41]. Instead, we observed differences in plasmatic viral load. Omicron variant was found to be linked to a significantly higher viral load compared with the others. To the best of our knowledge, there is a lack of data providing a comprehensive comparison across the various SARS-CoV-2 variants and plasmatic viral load.

The detection of SARS-CoV-2 RNAemia and its direct association with infectious viremia remains controversial [25], despite Jacobs et al. [24] demonstrated intact SARS-CoV-2 virions in plasma pellets from infected individuals following high-speed centrifugation. The presence of RNAemia is commonly associated to a severe disease, intensive care unit admission and death [27,42–47]. Additionally, patients with RNAemia were linked to a dysfunctional immune response and clinical complications [28,48,49]. For these reasons, different authors suggested that RNAemia may serve as a predictive marker for the COVID-19 condition [27,43–45]. Despite the extensive literature on the association between RNAemia and severe clinical outcomes, its occurrence remains ambiguous. Tang et al. [27] reported a positive rate of RNAemia at 34% (95% CI: 26%-43%), underscoring the potential influence of older age and the manifestation of severe/critical forms of COVID-19 on the presence of RNAemia. Within our cohort, RNAemia was observed in 61.9% of patients and 3.7% of those died. The exclusive recruitment of patients at high-risk of progression to severe COVID-19 could have influenced the high rate of patients with RNAemia.

Fifty-five percent of patients with RNAemia were vaccinated. Notably, our findings reveal that the clinical recovery time did not exhibit a significant difference between patients with and without RNAemia. In the existing literature, studies on RNAemia either utilized antiviral treatments or did not provide information on the specific treatment administered [42,43]. Therefore, in a previous work [50] and in our study, the favorable disease outcome is likely attributed to the early use of mAbs treatments and vaccination. Patients with SARS-CoV-2 RNAemia at the onset of symptoms had higher viral loads in NPS compared to those without RNAemia, contrary to what was reported by Kawasuji et al. [43]. Importantly, following treatments, viral loads similarly declined in both groups, indicating the beneficial impact of mAbs therapy on expediting viral clearance. In our study, an association between the presence of RNAemia and the Omicron variant was identified, but the limited number of patients infected with Omicron restricts the use of more robust statistical methods for a more detailed assessment. Data on Omicron are limited because antivirals (i.e., Molnupiravir, Nirmatrelvir/Ritonavir, and Remdesivir) have been approved for use in therapy for high-risk patients with mild-moderate COVID-19, proving effective against SARS-CoV-2 infection and demonstrating better patient compliance compared to mAbs [51,52].

## Conclusion

In the last decades, mAbs have emerged as therapeutic agents for cancer, immunological disorders, metabolic diseases and also for infectious diseases. In this context, mAbs play a crucial role in managing conditions such as emerging viral epidemics, where prophylactic drugs are often lacking, establishing themselves as a viable alternative to traditional antivirals and antibacterials.

During the COVID-19 pandemic, the rapid emergency approval and use of mAbs in a high-risk context provided valuable insights into their safety and clinical efficacy in real-life.

In conclusion, although anti-SARS-CoV-2 mAbs are no longer used in clinical practice due to their reduced efficacy against currently circulating variants, our real-life data underscores their significant clinical benefits, including reducing viral load in both NPS and plasma with minimal side effects. Importantly, these therapeutic approaches have the potential to positively impact clinical outcomes even in frail patients, allowing for a tailored management of COVID-19.

In a pandemic scenario, preparedness and rapid response are essential, and utilizing every available resource is key to support public health system. In future pandemics, this experience with mAbs can guide the rapid development and deployment of effective treatments, ultimately improving patient outcomes and public health responses.
